# The Multifactorial Causes of Down Syndrome During Pregnancy: A Narrative Review of Genetic, Environmental, and Maternal Influences

**DOI:** 10.1002/hsr2.71615

**Published:** 2025-12-04

**Authors:** Md. Sohel Rana, Mst. Morium Parvin, Md. Tariqul Islam

**Affiliations:** ^1^ Department of Biochemistry and Biotechnology Khwaja Yunus Ali University Sirajganj Bangladesh

**Keywords:** chromosomal nondisjunction, Down syndrome, genetic polymorphisms

## Abstract

**Background and Aims:**

Chromosomal nondisjunction is the primary etiology of Down syndrome; however, genetic variations, environmental causes, and maternal etiologies also contribute to a great extent. The objective of this review is to evaluate these multifaceted factors during pregnancy.

**Methods:**

A comprehensive review of the literature of research and epidemiology was performed to examine the environmental factors, genetic factors, and clinical variables that influence Down syndrome. The research concludes with a discussion of new prenatal testing methods, the danger presented by increased maternal age, and how such issues impact the affected families.

**Results:**

The genetic polymorphisms were associated with a higher rate of chromosomal nondisjunction, particularly in the genes for folate metabolism, including MTHFR, RFC1, and MTHFD. These factors like education level of the mother, income, and access to prenatal care can raise or lower Down syndrome. People born with problems, like heart problems or problems with their intestines, tend to have problems with their bodies and minds also. The article also mentions how hard it is for parents to deal with the emotional weight of having a prenatal diagnosis and how common this condition is in different racial and cultural groups.

**Conclusion:**

Down syndrome is still a complicated illness caused by genetics, the environment, and the mother. With enhanced prenatal screening to detect problems before birth, families still encounter moral and ethical dilemmas. We need to learn more through further research on genetic modifiers, how the environment affects them, and how they affect health in the long run. A deeper understanding of these concerns will make prenatal counseling more robust and more effective treatment procedures for individuals with Down syndrome.

## 1 Introduction

Down syndrome, or trisomy 21, is a genetic condition that results from the presence of one extra copy of chromosome 21. The disorder occurs primarily as a result of meiotic segregation mistakes in the chromosomes, 90% of which are the result of maternal nondisjunction [1]. The high genomic density of chromosome 21 renders it prone to segregation mistakes during meiosis. Down syndrome is the most common chromosomal condition that has been diagnosed by clinical observation and occurs in about 1 in 800 births worldwide [2]. Down syndrome is the leading genetic cause of intellectual disabilities [3]. Down syndrome occurs in individuals from all ethnic and racial backgrounds and is not restricted to a particular socioeconomic class [4]. Exposures to the environment, such as pollution, overpopulation, and deforestation, have been associated with several medical conditions, including pregnancy outcomes like Down syndrome. Yet, there are more investigations that need to be conducted to determine their direct contribution to risk for trisomy 21 [5]. Down syndrome is more common with advancing maternal age. For instance, the risk that a 20‐year‐old woman will give birth to a child with Down syndrome is 1 in 1980, but if the woman is 36 years old, the risk rises to 1 in 267. Due to this risk factor, pregnant women aged 35 and above are tested for Down syndrome automatically [4]. According to a recent Glasgow study, 1.2 out of every 1000 pregnancies result in Down syndrome. Of them, 19.1% were terminations after prenatal diagnosis, 29% were stillbirths, and 78.1% were live births. Abortion without provocation remains the most common outcome of Down syndrome pregnancies [6]. When aneuploidy is detected through prenatal diagnostic testing, couples must decide whether to continue or terminate the pregnancy. The growing use of prenatal diagnosis and advancements in prenatal screening have led to an increasing number of fetal abnormalities being identified early in pregnancy. While most parents who undergo prenatal testing will be relieved to find that their child does not have the condition the test was designed to detect, some parents will face the diagnosis of a fetus with abnormalities. With limited therapeutic options available, these parents must decide whether to continue the pregnancy, potentially delivering an infant with significant disabilities or limited survival prospects, or to consider pregnancy termination [7].

## 2 Methodology

This narrative review was conducted through a comprehensive search of existing literature and epidemiological studies. The following databases were used to identify relevant studies: PubMed, Google Scholar, Scopus, and Web of Science. The search was conducted using a combination of keywords including “Down syndrome,” “trisomy 21,” “chromosomal nondisjunction,” “folate metabolism,” “genetic polymorphisms,” and “environmental factors.” Studies published in English between 2000 and 2024 were considered for inclusion.

The inclusion criteria were as follows: studies focused on the genetic, environmental, and maternal risk factors for Down syndrome; studies that provided quantitative or qualitative data on the prevalence, risk factors, or clinical outcomes associated with Down syndrome; peer‐reviewed journal articles, clinical trials, and meta‐analyses [8, 9].

Exclusion criteria included: studies that did not focus on Down syndrome or its causes; articles not published in peer‐reviewed journals or lacking a clear methodology. The search was limited to studies published in English and included both human and animal studies where relevant to understanding human conditions. After initial screening, studies were included in the review, providing insights into the multifactorial causes of Down syndrome during pregnancy.

Statistical analysis and reporting were conducted in accordance with the guidelines proposed, and the SAMPL (Statistical Analyses and Methods in the Published Literature) guidelines [10, 11]. As this study is a narrative review, no original statistical analyses were performed. Where applicable, continuous variables in source studies are presented as mean ± standard deviation (SD) or median and interquartile range (IQR). Categorical variables are reported as frequencies and percentages with corresponding numerators and denominators. Odds Ratios (ORs) and Confidence Intervals (CIs) are cited as reported in the original publications. All *p* values are two‐sided and presented exactly as in the source studies [6, 12, 13].

## 3 Common Causes of Down Syndrome

### 3.1 Maternal Recombination Errors

Maternal recombination on chromosome 21 is a major, stage‐specific determinant of nondisjunction risk, with the strongest evidence showing that the placement of exchanges—rather than their overall number—drives errors in oocytes [14, 15]. In meiosis I (MI), trisomy 21 risk rises when there is no detectable crossover or when a single crossover is positioned telomerically on 21q, a pattern that predisposes to misorientation independent of maternal age [14, 15, 16]. During meiosis II (MII), risk is elevated for a single pericentromeric crossover, and this association is greater with older oocytes, as predicted by age‐linked loss of cohesion and spindle‐checkpoint integrity [14, 15, 16]. Analyses of hotspot usage suggest MI mistakes are overwhelmingly misplacement of exchanges, whereas MII mistakes show reduced correlation with LD‐defined hotspots within the centromeric region—implicating local chromatin/epigenetic environment in access to recombination [16]. Another line of evidence is that many females will be ovarian mosaics for trisomy 21 (≈0.2%–0.9% T21 cells in fetal ovaries), providing a biological substrate liable to be susceptible to distortions of recombination configurations and providing an explanation for the maternal‐age effect by selection and survival of trisomic oocytes throughout the ovarian lifespan [17]. Together, these concurrent reports render recombination placement a mechanistic aspect of maternal trisomy 21 risk and justify the inclusion of a dedicated subsection, “Maternal Recombination Errors,” placed before the genetic predisposition section to prime the reader for subsequent discussion of PRDM9, MCM9, and DNMT3B [14, 15, 16, 17].

### 3.2 Gene Variant Association

**Figure 1:**
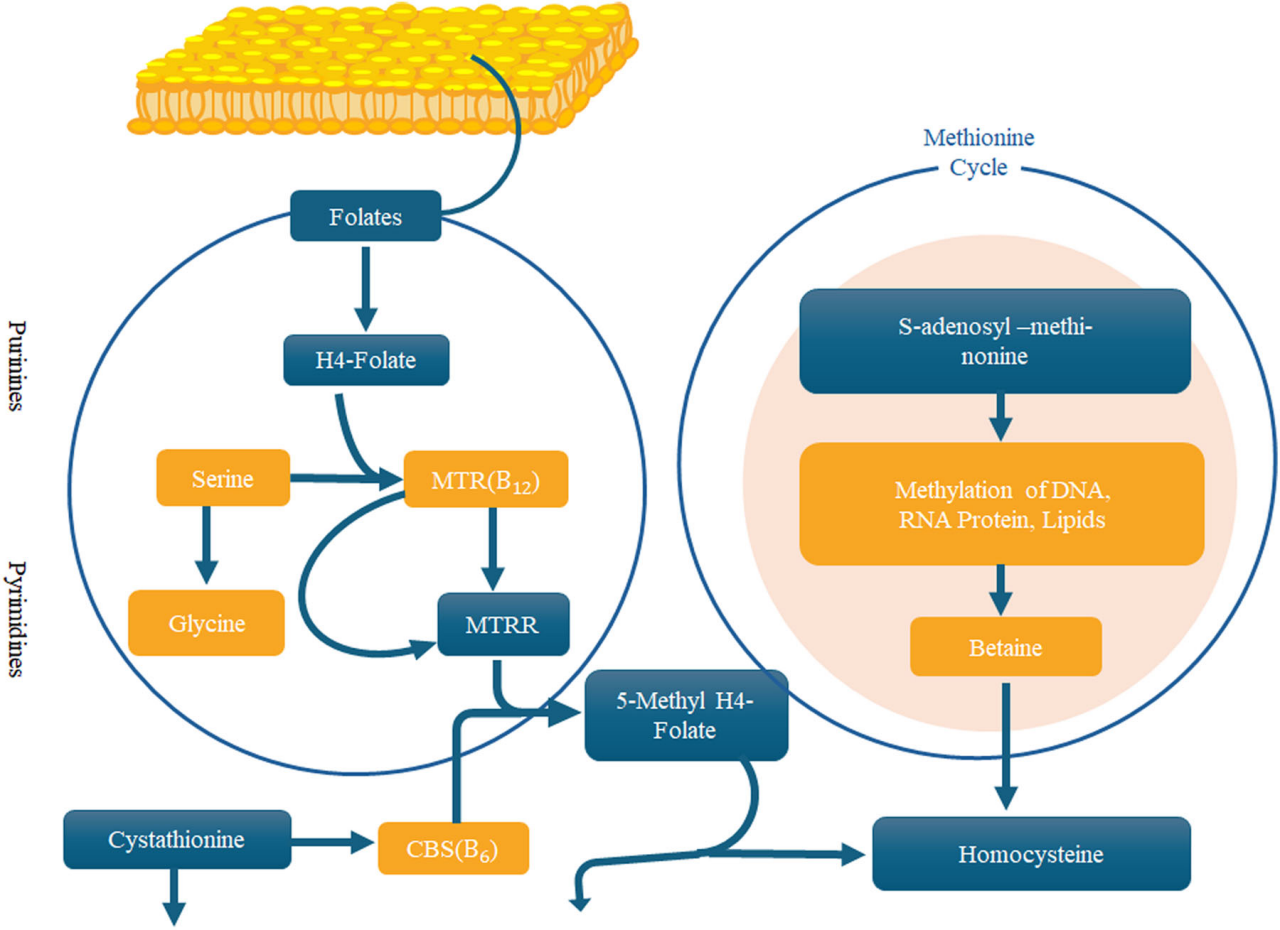
| Interaction between homocysteine and folate metabolism pathways in mothers with Down syndrome pregnancies. This figure illustrates how polymorphisms in MTHFR, RFC1, and MTHFD genes influence folate metabolism, DNA methylation, and chromosomal stability, leading to increased risk of chromosomal nondisjunction and trisomy 21.

The genetic mechanism evaluates how different genetic changes in mothers within the homocysteine‐folate pathway are related to the risk of offspring affected by Down syndrome. The MTHFR A1298C and RFCA A80G mutations are associated with elevated risk for Down syndrome, particularly in mothers aged ≥ 34 years at conception [1, 8]. Gene–gene interaction involving MTHFR, RFC1, and MTHFD genes showed significant positive associations [1] (Figure 1). When examining the relationship between maternal gene variants involved in folate metabolism and the likelihood of having children with Down syndrome. In the folate pathway, variants in folate‐metabolizing enzymes (MTHFR and MTRR) influence folate metabolism, DNA methylation, and chromosomal stability, which could contribute to Down syndrome risk [8].

Polymorphisms in genes involved in homocysteine and folate metabolism can affect folate balance and cellular methylation (Figure 1). One key enzyme, methylenetetrahydrofolate reductase (MTHFR), helps convert 5,10‐methylenetetrahydrofolate to 5‐methyltetrahydrofolate, which donates methyl groups for converting homocysteine (Hcy) into methionine (Met). Methionine is crucial for making S‐adenosylmethionine (SAM), the main methyl donor for DNA, protein, and lipid methylation. Two common MTHFR gene mutations, 677T and A1298C, reduce MTHFR activity, leading to higher folic acid needs and potentially affecting DNA methylation [8]. Recent research has identified several maternal genetic polymorphisms beyond the folate metabolism pathway that contribute to the risk of Down syndrome. A large case–control study demonstrated that novel variants in DNMT3B and RFC1 are strongly associated with meiosis II nondisjunction, independent of maternal age, thereby establishing DNMT3B as an additional maternal risk gene alongside folate regulators [18]. Similarly, variation in the PRDM9 zinc finger domain, a recombination regulator, has been shown to increase the frequency of maternal meiosis I nondisjunction without recombination on chromosome 21q, highlighting the critical role of recombination placement in trisomy 21 [19]. In addition, polymorphisms in MCM9, a DNA replication regulator, were found to predispose mothers to reduced recombination and meiotic errors during meiosis I, underscoring the importance of replication‐linked processes in chromosomal segregation [20]. A broader synthesis further emphasizes that maternal polymorphisms affecting recombination control, chromatin regulation, and segregation fidelity collectively shape nondisjunction risk, expanding the genetic landscape well beyond folate metabolism genes [21].

Recent GWAS and EWAS have identified maternal genetic variants (e.g., REC8, SMC1β, BUB1) that contribute to trisomy 21 risk [21, 22, 23, 24]. Recent genome‐wide association studies have provided robust evidence supporting the role of maternal variants in REC8 and SMC1β in meiotic nondisjunction leading to trisomy 21 [25]. Moreover, maternal epigenetic aging signatures like DNA methylation clocks and histone modifications are also being developed as biomarkers for trisomy 21 [16, 17, 18, 19, 26]. DNA hypomethylation in centromeric regions, which may result from folate deficiency or MTHFR polymorphisms, deranges chromosomal segregation [23, 24, 27, 28]. Maternal epigenetic aging, including methylation clocks and histone modifications, is a critical contributor to trisomy 21 risk [23, 24, 27, 28].

The enzyme methionine synthase (MTR) also helps convert Hcy to Met, supported by methionine synthase reductase (MTRR) [8]. Mutations in these genes (A2756G in MTR and A66G in MTRR) can disrupt methylation, especially in people with two copies of the A2756G variant. Another enzyme, cystathionine‐beta‐synthase (CBS), converts Hcy to cystathionine. The 844ins68 polymorphism in CBS, found in about 10% of people, can lead to lower homocysteine levels, especially after methionine intake. A mutation in the reduced‐folate carrier gene (RFC1), A80G, slightly lowers plasma folate levels, though the effect isn't significant [8]. Lastly, a G1958A polymorphism in the methylenetetrahydrofolate dehydrogenase (MTHFD) gene affects an enzyme that produces folate derivatives needed for DNA and nucleotide synthesis. While individual polymorphisms did not show significant associations, their combined effects substantially increase risk. This highlights the multifactorial nature of Down syndrome, involving genetic predisposition, advanced maternal age, and environmental interactions. Moreover, other genetic causes include translocations and mosaicism, which contribute to variations in phenotype expression [1, 8]. The MTHFR enzyme, which is crucial for converting 5,10‐methylene THF to 5‐methyl THF, is impaired in individuals with the C677T and A1298C polymorphisms. This leads to reduced SAM production and elevated homocysteine, impairing DNA methylation, disrupting epigenetic regulation, and destabilizing chromosomal integrity [29, 30, 31, 32] (Figure 1).

### 3.3 Consanguineous Marriage as a Genetic Risk Factor

Consanguineous marriage has emerged as an important maternal risk factor for Down syndrome, particularly in populations where such unions are culturally prevalent [33]. In a study, it was reported that parental consanguinity was significantly associated with maternal meiosis II nondisjunction at younger maternal ages, even in the absence of detectable recombination errors, suggesting that increased omozygosity may compromise chromosomal segregation fidelity [34]. Complementing this genetic evidence, a clinic‐based cohort of 221 individuals with Down syndrome in the United Arab Emirates found that 38.6% of families reported consanguinity, reflecting the high prevalence of the practice in this region [33]. While their analysis did not establish consanguinity as a direct determinant of DS incidence, the study emphasized its potential contribution to the spectrum of co‐occurring congenital conditions, especially cardiac malformations, through excess homozygosity [33]. Importantly, further demonstrated that maternal nondisjunction errors were overwhelmingly more frequent in consanguineous families, with a striking shift toward meiosis II errors (74% in CM vs. ~10% in NCM) [34]. Mothers in CM families were on average 4–5 years younger at conception than NCM mothers, indicating that consanguinity increases DS risk even at younger maternal ages [34]. Moreover, CM families displayed distinct recombination patterns, characterized by medial recombination placements rather than the typical telomeric or peri‐centromeric patterns observed in MI or MII errors [34]. Together, these findings underscore that consanguinity should be considered alongside maternal gene polymorphisms and advanced age as a significant factor predisposing to nondisjunction, particularly in Middle Eastern and South Asian populations, where the practice remains common [33, 34].

### 3.4 Environmental Influences

Environmental exposures to pollutants and toxins can cause epigenetic changes, including altered DNA methylation. These changes can increase the risk of trisomy 21 in older mothers. These environmental factors induce abnormal segregation of the chromosomes during meiosis and thereby contribute to the etiology of the increased risk of Down syndrome [21, 23, 35]. DNA hypomethylation in centromeres by either folate deficiency or polymorphism in MTHFR interrupts the segregation of the chromosomes, increasing the risk of nondisjunction [21, 23, 35]. The relationship between smoking and Down syndrome is one of contradiction and paradox. Other studies suggest that smoking may increase the risk for trisomy 21 by oxidative stress and interference with DNA methylation patterns, though others find no relationship. The inconsistencies may be due to variation in study design, population, and exposure timing [21, 35]. A better‐controlled, more stringent test of the dose–response relationship between smoking and risk for trisomy is needed to resolve these inconsistencies [21, 35]. Bisphenol A (BPA), an endocrine disruptor, was shown to influence chromosome stability. BPA can induce disturbances in meiotic segregation, leading to cell division mistakes. Degradation of the cohesin proteins during exposure to BPA may be impaired, supporting chromosomal missegregation [31, 32]. Exposure to BPA has also been linked with increased frequency of chromosomal abnormalities, including trisomy 21. This association underscores the need for environmental control to prevent exposure at early oocyte stages [31, 32].

### 3.5 Maternal Health

Maternal health disorders, including obesity and diabetes, have been linked with an elevated risk of trisomy 21. Maternal obesity is a risk factor for Down syndrome, possibly because it influences the balance of hormones and metabolic stress in pregnancy [35]. However, there are no uniform results that implicate an association of obesity and increased risk of trisomy 21. There may be confounding variables, such as maternal age and genetic predisposition [35]. Such studies, which control for these factors, are valuable in determining the exact contribution of obesity towards trisomy risk. These conditions may disrupt the normal cellular functions during meiosis and affect chromosomal segregation and thus increase the likelihood of nondisjunction. Pregnancy‐induced metabolic stress may also augment these risks [7, 23, 36].

### 3.6 Chromosomal Nondisjunction

**Figure 2:**
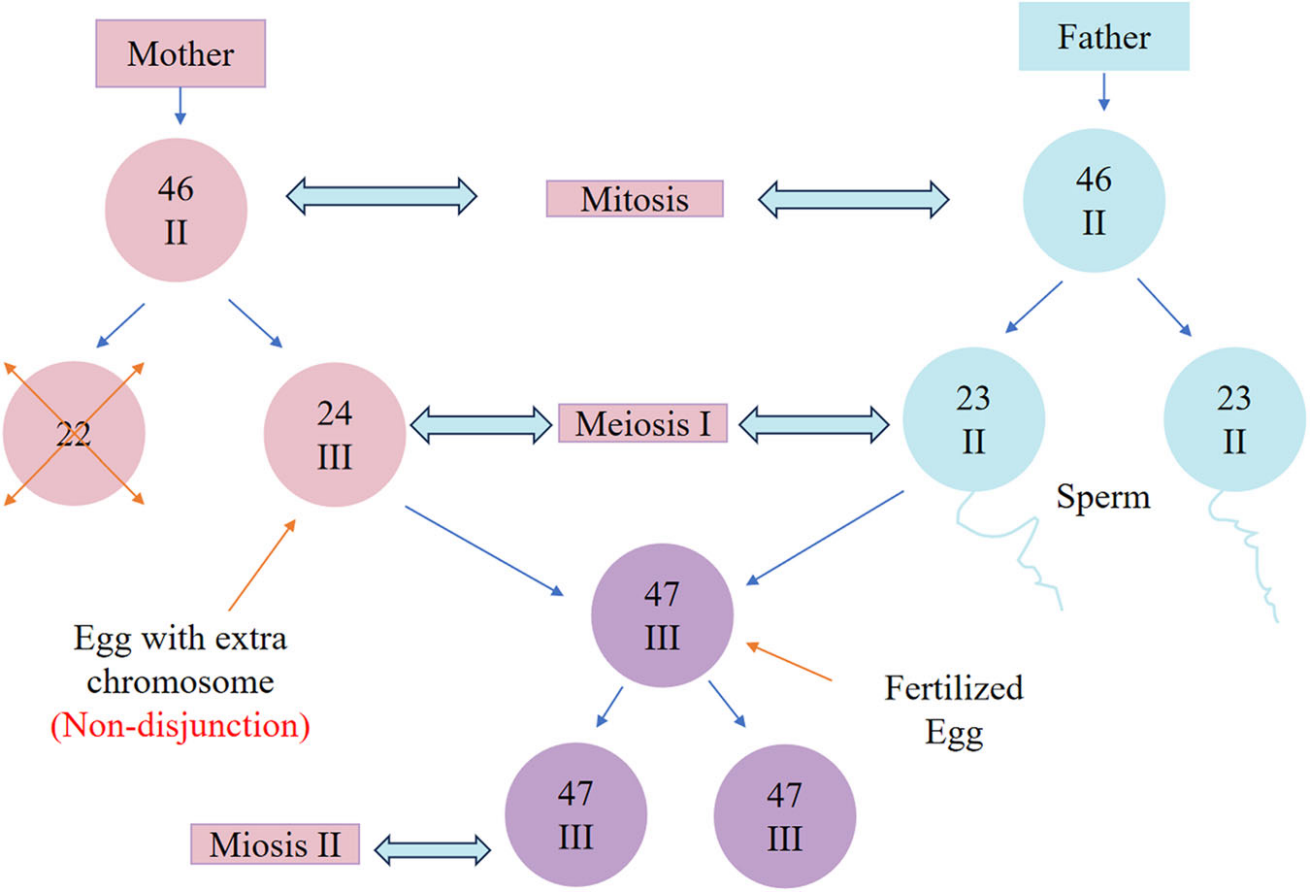
| Mechanisms of meiotic nondisjunction resulting in an additional copy of chromosome 21. The diagram shows how age‐related loss of cohesin proteins, recombination errors, and spindle assembly defects contribute to missegregation during oocyte meiosis, leading to Down syndrome.

Down syndrome (DS) is primarily caused by the presence of an extra chromosome 21 due to meiotic error during the formation of reproductive cells (meiosis) [1]. Down syndrome is highly associated with advanced maternal age as a result of meiotic division error. The mechanism involves the degradation of cohesin proteins (Figure 2), such as REC8 and SMC1β, necessary for sister chromatid cohesion in meiosis. With advancing age, the cohesion between such proteins is lost, most significantly during the prolonged phase of oocyte arrest [30, 31]. This impaired cohesion predisposes to nondisjunction and trisomy 21 (Figure 2). Studies showed that the decline in cohesin proteins resulted in an inability to properly align chromosomes, significantly raising the chance of aneuploidy during cell division [30, 31]. About 35% of cases result from such errors, mainly occurring in the mother's eggs. Most maternal errors happen during meiosis I (74.7%), while a smaller portion occurs in meiosis II (25.3%) [9]. The relatively small size and genomic composition of chromosome 21 increase its susceptibility to meiotic segregation errors, leading to Down syndrome [9]. While maternal errors are the most common cause, about 5%–10% of Down syndrome cases result from paternal non‐disjunction, where the father's sperm contributes the extra chromosome 21 [37].

Different chromosomes show varying rates of paternal trisomy. For example, all cases of 47, XYY (an extra Y chromosome) come from paternal errors, while 40%–50% of 47, XXY (Klinefelter syndrome) and trisomy 2 cases are also paternal [10]. However, paternal errors are much less common in 47, XXX and most autosomal trisomies and are rarely seen in trisomy 16. This variation in paternal trisomies might be due to chromosome‐specific differences in how errors occur in sperm formation [10]. Two possible reasons have been suggested: the rate of paternal errors might be similar across chromosomes, but maternal errors are much more frequent, making paternal contributions seem smaller. Genomic imprinting (the way genes are marked depending on whether they come from the mother or father) could affect which trisomic embryos survive. Errors in meiosis often involve problems with recombination, the process by which chromosomes exchange genetic material. Studies show that maternal errors in sex chromosomes and autosomes (like chromosomes 15, 16, 18, and 21) often have reduced recombination [10]. In some cases, there is no recombination at all. For example, nearly 70% of paternal 47, XXY cases result from an XY chromosome pair failing to recombine. Similarly, about 40% of maternal meiosis I trisomy 21 cases have no recombination, making them more prone to errors [10]. Although paternal factors are underexplored, some studies suggest that paternal age and genetic contributions may influence trisomy 21 outcomes [35, 38]. There is evidence that sex chromosomes and epigenetic modifications differ between male and female offspring with trisomy 21, which may influence phenotypic outcomes [28, 38, 39]. It is associated with accelerated biological aging, as shown by DNA methylation clocks, suggesting that trisomy 21 may be a form of segmental progeria [9, 40, 41]. With advancing maternal age, meiosis‐specific cohesin proteins like REC8 and SMC1β are progressively lost, particularly in the oocytes' prolonged arrest phase. This loss weakens sister chromatid cohesion, increasing the likelihood of meiotic nondisjunction and resulting in trisomy 21 [29, 30, 31, 32].

### 3.7 Epigenetics and Gene–Environment Interactions

**Figure 3:**
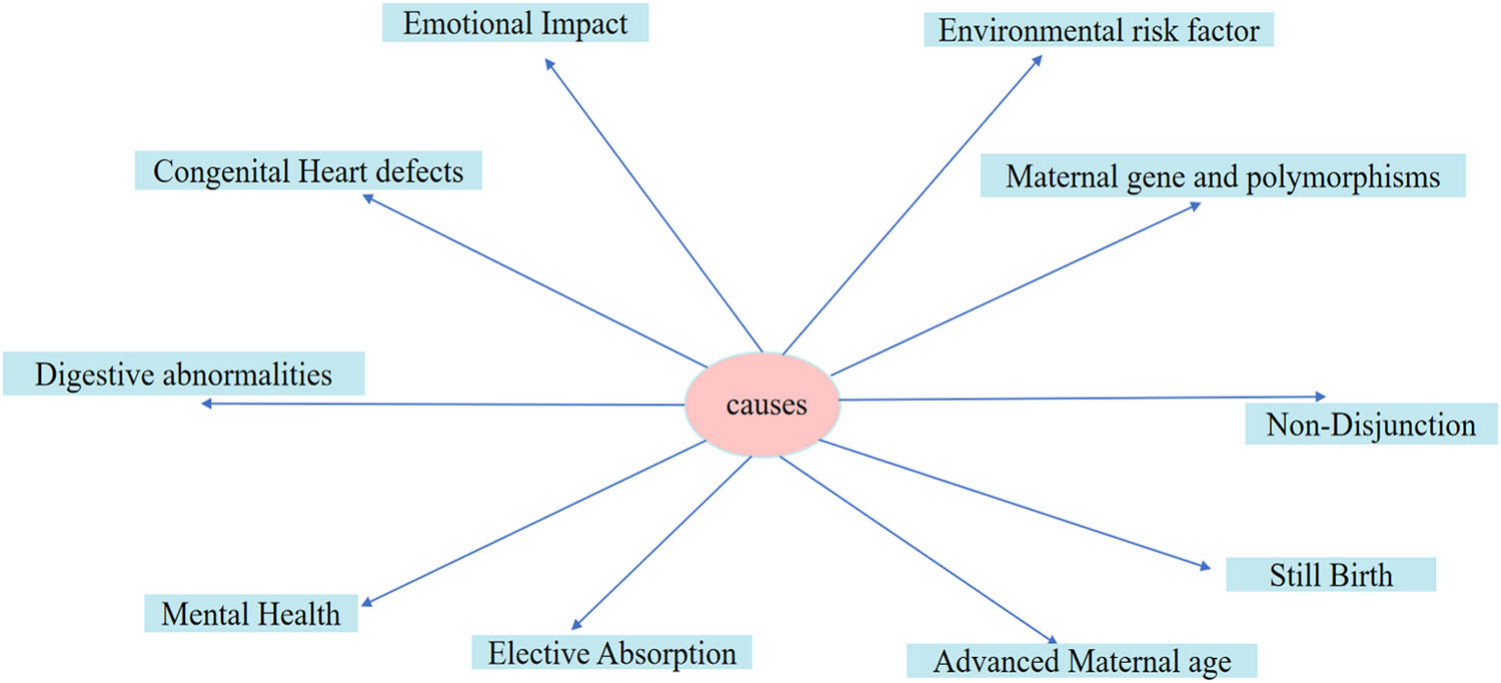
| Overview of genetic, environmental, and maternal factors contributing to Down syndrome development during pregnancy. The figure integrates pathways such as folate metabolism, environmental exposures (e.g., bisphenol A, smoking), maternal health conditions, and epigenetic modifications that disrupt chromosomal segregation.

Epigenetic modifications, including DNA methylation and histone modification, play an important role in chromosomal stability and segregation. Both predisposition and environmental factors (Figure 3) can influence these changes in the control system between genetic and nongenetic variables. They have thus added further complexity to the trisomy 21 risk equation [21, 30]. Maternal age, combined with genetic differences in genes related to folate metabolism such as MTHFR and RFC1, can change the body's epigenetic regulation. This disrupts normal chromosomal activity during meiosis, a crucial event for all egg cells that leads to the formation of all offspring from that egg. As a result, nondisjunction may occur more often, and Down syndrome will be more likely [21, 30].

Exposures to the environment, such as smoking, endocrine‐disrupting compounds such as Bisphenol A (BPA), and dietary insufficiencies (e.g., folate), can be accountable for these epigenetic alterations, thereby influencing the risk of Down syndrome [21, 30]. Studies showed that exposure to BPA, for example, impaired chromosomal stability by triggering the degradation of cohesin protein, a prerequisite for sister chromatid cohesion during mitosis. Additionally, DNA hypomethylation of centromeric regions has the potential caused by folate deficiency or genetic polymorphisms of folate‐metabolizing genes has been correlated with increased nondisjunction and meiotic errors [21, 30].

Maternal age‐related epigenomic alterations are also significant. Aging may result in the alteration of DNA methylation profiles and histone modifications, particularly in genes for chromosomal stability. Such epigenomic alterations might predispose older mothers to a greater risk of trisomy 21, and hence, consideration of epigenetic markers of aging is crucial while assessing risk. Understanding the molecular mechanisms of such interactions can aid in the development of more specific screening strategies and therapeutic interventions for Down syndrome [21, 30].

While the genetic contribution to the primary cause, epigenetic regulation, and variation in other genes, for example, Down syndrome Cell Adhesion Molecule (DSCAM) gene and amyloid precursor protein gene, can influence the severity of clinical presentation [11]. Faint evidence suggests contributions from maternal smoking, hormonal imbalance, and genetic polymorphism [11]. Environmental hazards such as cigarette smoking and occupational toxins have been associated with an increased risk of trisomy 21 (Figure 3), especially in advanced maternal age [13, 35, 38, 42]. Important pathways to trisomy 21 risk include breakdown of cohesin proteins, oxidative damage, and metabolic alterations to disrupt chromosome separation during meiosis. Gene × environment interactions, including exposure to environmental toxins and pollutants, also further erode meiotic integrity [22, 24, 35, 43]. A multifactorial framework that includes genetic factors, maternal age, epigenetic modifications, environmental exposures, and socioeconomic determinants explains the risk of trisomy 21 [22, 27, 44, 45]. Epigenetic modifications in trisomy 21, such as changes in DNA methylation, affect neurodevelopmental outcomes. They supposedly explain cognitive delay and neurodegeneration in people with Down syndrome [38, 40, 46, 47].

Maternal lifestyle exposures like alcohol use, cigarette smoking, and chewing/smokeless tobacco use have emerged as important environmental risk factors for Down syndrome [48, 49, 50]. These exposures can induce oxidative stress, folate cycle disturbances, and epigenetic changes that disrupt meiotic recombination and chromosome segregation [48, 50]. Evidence from Brazil shows that, even where alcohol and smoking were infrequently reported, they remain mechanistically relevant due to their role in nutritional imbalance and meiotic instability [50]. More critically, chewing/smokeless tobacco has been directly linked to elevated DS risk, with case–control studies showing an almost threefold increase in odds of DS births among users, independent of maternal age [2, 49]. This effect is amplified through interactions with maternal folate‐pathway polymorphisms, leading to reduced recombination and pericentromeric crossovers associated with meiosis II nondisjunction [49]. Given its high prevalence in Bangladesh and South Asia, chewing tobacco may represent a particularly significant regional risk factor, acting synergistically with genetic predispositions to increase nondisjunction risk [48, 50].

### 3.8 Emotional Impact

#### 3.8.1 Anxiety and Depression

Prenatal screening often raises concerns regarding fetal health and viability. Women undergoing Assisted Reproductive Technology (ART) often experience greater anxiety due to the prolonged journey to conception and additional risk factors related to maternal age [12]. The psychological burden of ART cycles and prenatal screening can contribute to prepartum depression. Moreover, prenatal attachment to the fetus often increases after receiving hormonal screening results, as older mothers are the most important known risk factor. Hypotheses also point to potential environmental and genetic influences impacting oocyte quality during maternal development or before conception [12].

Down syndrome pregnancies have higher rates of spontaneous loss, particularly detectable cases through prenatal screening. In prenatal screening, it has identified more Down syndrome pregnancies, contributing to trends in termination and the understanding of spontaneous loss rates. Improved antenatal screening methods have resulted in higher rates of prenatal diagnosis and pregnancy termination [12]. Elevated oxidative stress, such as reduced Nuclear Factor Erythroid 2‐Related Factor 2 (NRF2) activity and increased NAD(P)H Quinone Dehydrogenase 1 (NQO1), serves as a novel biomarker for trisomy 21 [13, 24, 45, 51]. Oxidative stress‐induced epigenetic modifications in maternal tissues could predict trisomy risk, supporting noninvasive screening strategies [13, 24, 45, 51, 52]. Maternal health conditions, such as pre‐eclampsia and metabolic stress, accelerate epigenetic aging, affecting fetal development and increasing trisomy risk, while using epigenetic clocks, like DNA methylation (DNAm) age, could help predict risks associated with advanced maternal age [9, 12, 21, 27].

## 4 Current Statistics and Trends in Down Syndrome

### 4.1 Prevalence of Down Syndrome

In one study, we saw that Down syndrome occurs approximately 1 in 732 live births in the US [13]. In another study, the prevalence for individuals who are 16 years old and above in the UK is approximately 5.9 per 10,000 of the general population. Life expectancy has improved significantly. Seventy‐five percent of individuals live to age 50, half reach 58.6 years, and a quarter survive until 62.9. However, these rates remain lower than those of the general population [6]. In Western Australia, the overall prevalence increased from 1.1 per 1000 births in 1980 to 2.9 per 1000 births in 2004, influenced by rising maternal age. Down syndrome affects 1 in 650–1000 live births [14].

### 4.2 Implication of Socioeconomic Condition on DS Birth

We saw in another analysis that about 997 Down syndrome cases and 1007 controls revealed that Socioeconomic Status (SES) is associated with an increased risk of Down syndrome, although the underlying mechanisms are not fully understood [15]. Among them 29% (289/997) of mothers without a high school education faced a 29% increased risk (OR: 1.29; CI:1.01–1.65) [odds ratio (OR), confidence interval (CI)] [15]. Families with annual incomes below $20,000 faced a 31% (310/997) increased risk (OR: 1.31; Cl: 1.02–1.68) [15]. A composite SES score demonstrated that the presence of four low‐SES elements was present in 20% (200/997) of pregnancies and nearly doubled the risk of Down syndrome pregnancy (OR: 1.31; CI: 1.30–3.01) [15].

Across the USA, a 30‐year geotemporospatial/causal analysis integrating socioeconomic and ethnocultural covariates with maternal substance‐use exposures showed that Down syndrome rates are socially patterned, with cannabis measures emerging as significant predictors and higher rates where cannabis was not illegal [53]. Maternal occupation was also linked to stage‐specific nondisjunction risk: production work increased MII risk, scientist and food‐service jobs increased MI risk, and within production jobs, solvent exposure was a strong predictor of MI nondisjunction [35]. In India's Sundarban—an extremely low‐SES setting—mothers showed a distinct meiotic‐error profile with a higher proportion of MII errors and fewer achiasmate MI events, indicating elevated nondisjunction risk even when recombination occurs [54]. Together, these findings strengthen the *Socioeconomic Prevalence* section by demonstrating that DS epidemiology is influenced by socioeconomic/ethnocultural context, occupation‐linked chemical exposures, and substance use [51]. That poverty‐driven environments can reshape meiotic mechanisms beyond maternal age effects [35, 53, 54].

### 4.3 Co‐Occurring Health Conditions in Down Syndrome

**Figure 4:**
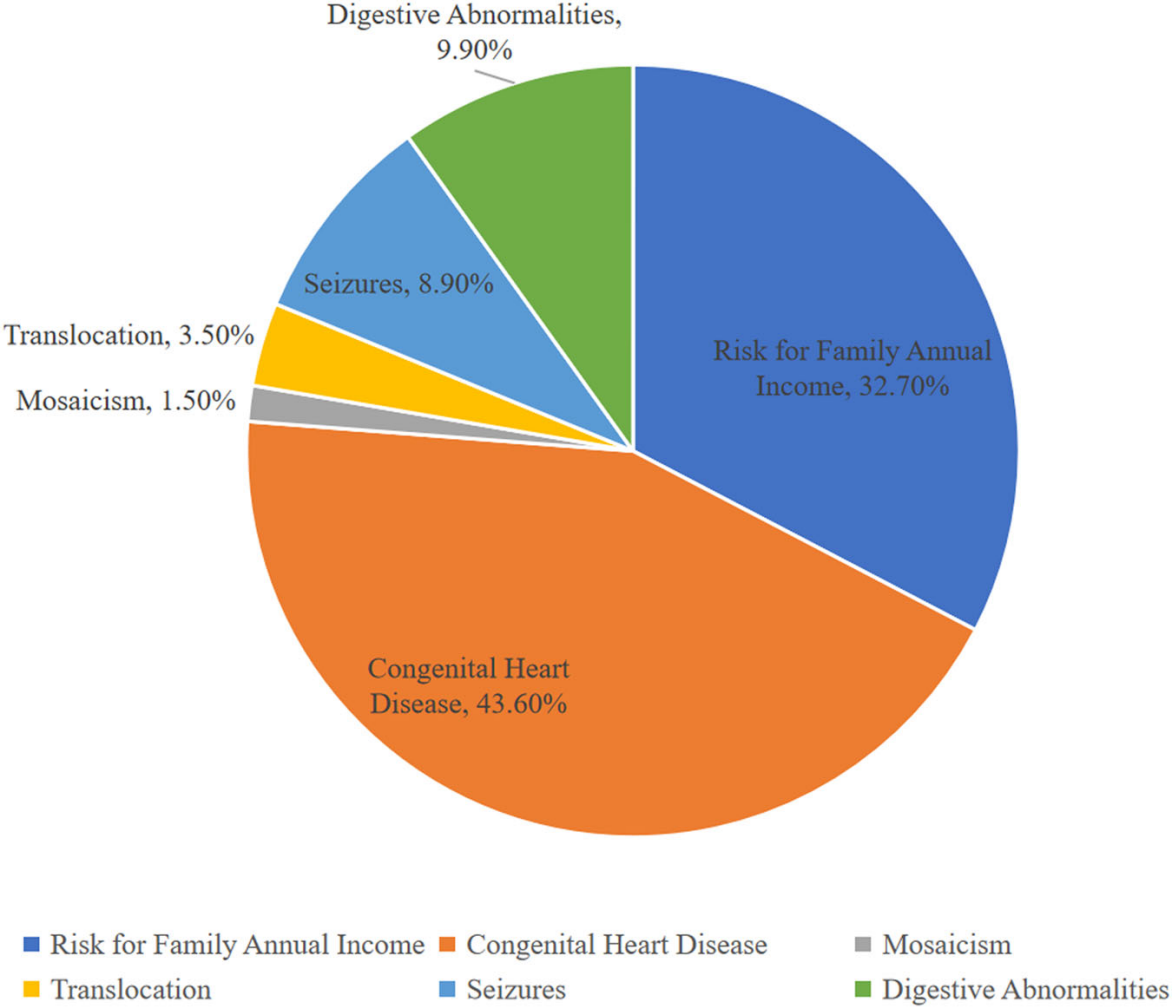
| Prevalence of associated health complications and distribution of genetic variants observed in Down syndrome. This chart depicts the frequency of congenital heart defects, digestive abnormalities, and neurodevelopmental conditions, as well as the relative contribution of genetic polymorphisms to disease susceptibility.

Individuals with Down syndrome (DS) commonly present with a broad, lifelong spectrum of co‐occurring conditions [2, 55, 56]. About 50% have congenital heart disease (typically AVSD, VSD, or ASD), and there is increased susceptibility to autoimmune disorders—especially thyroid disease (congenital hypothyroidism and autoimmune thyroiditis) and celiac disease [55] (Figure 4). Hematologic complications are prominent: ~10% of neonates develop transient abnormal myelopoiesis (with 20%–30% risk of progression to AML), and 2%–3% overall develop leukemia; concomitant immune dysregulation contributes to recurrent respiratory infections [2]. Across the lifespan, developmental delays and neurobehavioral challenges (intellectual disability, ASD features, psychiatric disorders, epilepsy) are common, and Alzheimer‐like neuropathology emerges almost universally after age 40 [2, 55]. Nutritional and metabolic factors compound these risks: children with DS frequently show deficiencies in folate/B‐vitamins, antioxidants, zinc, and selenium, alongside obesity driven by a lower resting metabolic rate, altered leptin signaling, reduced activity, and carbohydrate‐dense diets—factors that heighten oxidative stress, impair thyroid function, and worsen developmental outcomes [56].

Early, systematic surveillance and multidisciplinary care—that is, cardiac screening, endocrine/immune monitoring, infection prevention, neurodevelopmental intervention, and individualized nutrition/weight management—can forestall morbidity and improve quality of life [2, 55, 56].

44% (440/1000) of individuals with Down syndrome have congenital heart disease. Atrioventricular septal defects are the most common (45%, 198/440), followed by ventricular septal defects (35%, 154/440) and patent ductus arteriosus (7%, 31/440). Apart from that, we noted 3%–4% (30–40/1000) due to translocation, 1%–2% (10–20/1000) because of mosaicism [13], and 10% (100/1000) have gastrointestinal defects [9]. It can also lead to a range of physical, intellectual, and behavioral characteristics. Approximately 65% (650/1000) of individuals experience sleep disorders, and around 50% (500/1000) of adults develop hypothyroidism or Hashimoto's disease [13]. Moreover, about 8% of individuals encounter seizures during their lifetime. Additionally, autism spectrum disorder is observed in 7%–16% (70–160/1000) of cases [13].

**Figure 5:**
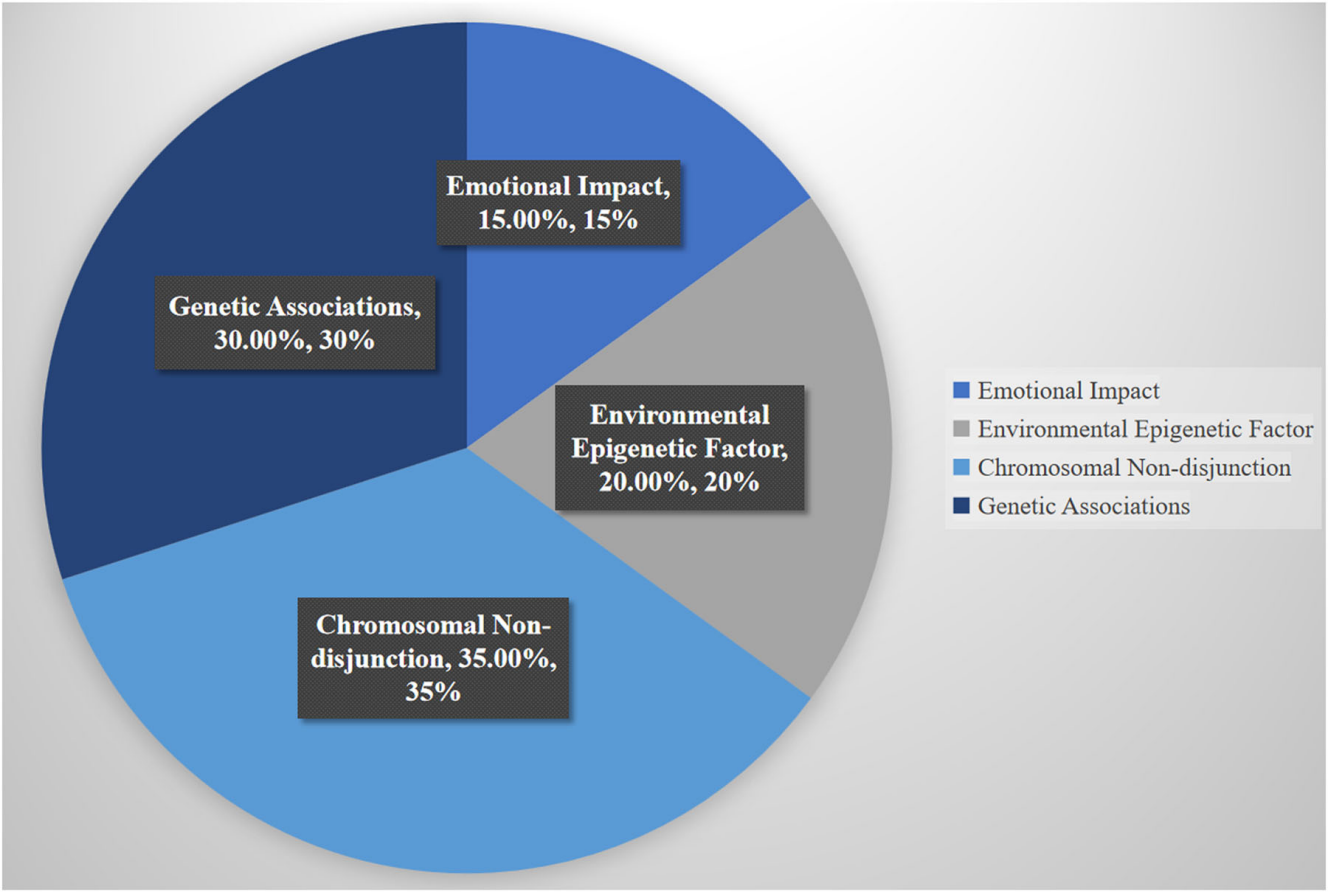
| Contributing factors and psychosocial impacts associated with Down syndrome diagnoses. The illustration summarizes risk factors, including advanced maternal age and socioeconomic status, alongside emotional challenges faced by families, such as anxiety, depression, and decisions regarding pregnancy continuation.

Table 1 lists various health conditions and their prevalence among individuals with Down syndrome. Sleep disorders affect 65%, and hypothyroidism or Hashimoto's disease occurs in 50%. Congenital heart defects are common, with atrioventricular septal defects in 45% and patent ductus arteriosus in 7%. Additionally, autism spectrum disorder is observed in 7%–16% (70–160/1000), while mental health issues affect 23.7% (237/1000). Socioeconomic factors also play a role, as 29% of mothers of individuals with Down syndrome have less than a high school education, potentially impacting healthcare access and outcomes (Figure 5; Table 1).

**Table 1:** | This table presents the proportion of diseases found in individuals with Down syndrome across different life stages (both children and adults).

Health condition	Percentages
Mother with less than a high school education	29% (289/997)
Atrioventricular septal defects	45% (198/440)
Patent ductus arteriosus	7% (31/440)
Sleep disorder	65% (650/1000)
Hypothyroidism or Hashimoto's disease	50% (500/1000)
Autism spectrum disorder	7%–16% (70–160/1000)
Mental ill health	23.7% (237/1000)

### 4.4 Racial/Ethnic Variability

Down syndrome is more prevalent among Hispanic mothers compared to non‐Hispanic White and Black mothers. The prevalence ratio for Hispanic mothers is 1.12, indicating a slightly higher occurrence than in non‐Hispanic White mothers. In contrast, non‐Hispanic Black mothers have a lower prevalence ratio of 0.77 when compared to non‐Hispanic White mothers [9].

### 4.5 Prenatal Diagnosis and Termination Rates

Pregnancy termination rates following a Down syndrome diagnosis range from 61% to 93%, influenced by various demographic, cultural, and personal factors [7]. Religious, moral, and personal beliefs play a significant role in the decision to continue the pregnancy after such a diagnosis. Approximately 33% (330/1000) of mothers had predetermined, before pregnancy, that they would not consider termination under any circumstances [7]. In contrast, 67% (670/1000) of mothers made their decision regarding pregnancy continuation only after receiving a diagnosis or screening result [7].

### 4.6 Mental Health

The point prevalence of any mental health issues (excluding specific phobias) in adults with Down syndrome is 23.7% [15]. The most common mental health issues include problem behaviors (10.2%), dementia (70%), and affective disorders such as depression (2.7%) [15]. Moreover, over 2 years, the incidence of new mental health conditions is 14.9%, with depressive episodes and dementia/delirium being the most prevalent [15]. Interestingly, the prevalence of mental health issues is lower in adults with Down syndrome compared to those with intellectual disabilities from other causes, suggesting the presence of potential biological protective factors [15].

## 5 Challenges and Gaps in Down Syndrome Research

It studies differences in genetic effects across populations, likely due to distinct genetic backgrounds and dietary habits; further research in diverse populations is required. Advanced maternal age and altered recombination patterns are established risk factors; the influence of environmental exposures and genetic modifiers requires further exploration. The reason for variability in Down syndrome‐associated birth defects, such as congenital heart disease and gastrointestinal anomalies, is poorly understood. There is a lack of large‐scale, ethnically diverse, population‐based studies on Down syndrome prevalence beyond infancy to adulthood, particularly to understand survival rates, morbidity, and long‐term healthcare needs. These insights can inform improved prenatal counseling, personalized risk screening methods, and public health policies beyond just maternal age, considering epigenetic and environmental factors [12, 44, 45, 57]. Notable research gaps include the lack of ethnic diversity in trisomy 21 studies, underexplored paternal factors, and insufficient investigation into new environmental toxins [13, 24, 28, 35, 42]. The need for multi‐omic, longitudinal studies to better link epigenetic markers with clinical outcomes remains a priority [13, 24, 28, 35, 42]. Identification of biomarkers like NRF2 and CSE, along with DNA methylation markers, offers new avenues for improving prenatal screening and early diagnosis of trisomy 21 [12, 13, 45]. Longitudinal research incorporating genetic, epigenetic, and environmental data is needed to elucidate trisomy 21's multi‐dimensional risk factors [13, 24, 57].

## 6 Conclusion

Down syndrome remains a complex condition influenced by genetic, chromosomal, and environmental factors, with advanced maternal age being a major risk determinant. Its prevalence and associated conditions vary significantly across populations, reflecting the interplay of biological, socioeconomic, and cultural factors. While advances in screening and diagnosis have enhanced early detection, they have also introduced ethical and emotional challenges for families. Future research must focus on bridging knowledge gaps related to genetic modifiers, environmental influences, and long‐term health outcomes. Addressing these areas will aid in developing more comprehensive care strategies and improving the standard of living for people with Down syndrome and their families.

## 7 Clinical Implications

The findings of this review have significant clinical relevance to early intervention planning, genetic testing, and prenatal counseling. With knowledge of the multifactorial etiology of Down syndrome and the role of genetic, environmental, and epigenetic factors, clinicians can offer more tailored counseling to potential parents, especially those at higher risk through determinants such as advanced maternal age, family backgrounds, and environmental exposure [28, 36, 45]. Healthcare providers can employ this information to tailor risk evaluation to patients based on both genetic screening and environmental status. Examples of maternal disease, such as obesity and diabetes, and environmental factor exposure, like smoking or BPA, can be incorporated into risk evaluation to increase the likelihood of predicting the occurrence of Down syndrome [21, 28, 45]. Tailored prenatal counseling will allow parents to make decisions regarding prenatal screening, testing, and care planning [21, 45].

Second, the environmental and epigenetic causes highlighted in this review suggest the need for the application of early diagnostic tools able to detect epigenetic markers with a correlation to trisomy 21 [28, 30]. These tools could be used along with traditional genetic tests to enhance the accuracy of early diagnosis, particularly among older mothers whose increased risk is due to age‐dependent epigenetic changes of their oocytes [8, 28, 30, 36].

The addition of genetic counseling, maternal health management, and environmental risk factor consideration will enable a comprehensive model of prenatal care. With the integration of these multifaceted components, mother‐child outcomes are improved as families are more effectively served with the most up‐to‐date information and alternatives [8, 28, 36, 45]. Prenatal counseling should incorporate genetic, environmental, and maternal age‐related factors to assess Down syndrome risk. Discussing genetic testing, including noninvasive prenatal testing (NIPT), along with lifestyle factors such as maternal age and exposure to environmental toxins, is crucial for informed decision‐making [22].

## Author Contributions

**Md. Sohel Rana and Mst. Morium Parvin:** review, data collection, formal analysis, preparing the draft. **Md. Tariqul Islam:** supervision, formal analysis, project administration, visualization, and investigation.

## Ethics Statement

The authors have nothing to report.

## Conflicts of Interest

The authors declare no conflicts of interest.

## Transparency Statement

The corresponding author, Md. Tariqul Islam, affirms that this manuscript is an honest, accurate, and transparent account of the study being reported; that no important aspects of the study have been omitted; and that any discrepancies from the study as planned (and, if relevant, registered) have been explained.

## Data Availability

This article is a narrative review based on publicly available literature. All data supporting the findings are derived from the cited sources and can be accessed through the respective references.

## References

[1] E. Zintzaras , “Maternal Gene Polymorphisms Involved in Folate Metabolism and Risk of Down Syndrome Offspring: A Meta‐Analysis,” Journal of Human Genetics 52 (2007): 943–953, 10.1007/s10038-007-0202-x. 17934692

[2] M. J. Bull , “Down Syndrome,” New England Journal of Medicine 382 (2020): 2344–2352, 10.1056/NEJMra1706537. 32521135

[3] A. P. C. Brandalize , E. Bandinelli , P. A. D. Santos , and L. Schüler‐Faccini , “Maternal Gene Polymorphisms Involved in Folate Metabolism as Risk Factors for Down syndrome Offspring in Southern Brazil,” Disease Markers 29 (2010): 95–101, 10.3233/DMA-2010-0731. 21045269 PMC3835528

[4] H. Choi , M. van Riper , and S. Thoyre , “Decision Making Following a Prenatal Diagnosis of Down Syndrome: An Integrative Review,” Journal of Midwifery & Women's Health 57 (2012): 156–164, 10.1111/j.1542-2011.2011.00109.x. 22432488

[5] M. Donohoe , “Causes and Health Consequences of Environmental Degradation and Social Injustice,” Social Science & Medicine 56 (2003): 573–587. 12570975 10.1016/s0277-9536(02)00055-2

[6] D. Mantry , S. A. Cooper , E. Smiley , et al., “The Prevalence and Incidence of Mental Ill‐Health in Adults With Down syndrome,” Journal of Intellectual Disability Research 52 (2008): 141–155, 10.1111/j.1365-2788.2007.00985.x. 18197953

[7] E. Hurford , A. Hawkins , L. Hudgins , and J. Taylor , “The Decision to Continue a Pregnancy Affected by Down syndrome: Timing of Decision and Satisfaction With Receiving a Prenatal Diagnosis,” Journal of Genetic Counseling 22 (2013): 587–593, 10.1007/s10897-013-9590-6. 23604903

[8] A. H. Bittles , C. Bower , R. Hussain , and E. J. Glasson , “The Four Ages of Down syndrome,” European Journal of Public Health 17 (2007): 221–225, 10.1093/eurpub/ckl103. 16857692

[9] N. El Hajj , M. Dittrich , J. Böck , et al., “Epigenetic Dysregulation in the Developing Down syndrome Cortex,” Epigenetics 11 (2016): 563–578, 10.1080/15592294.2016.1192736. 27245352 PMC4990229

[10] C. Sook Hong , “Studies of Association of Environmental Risk Factors in Down Syndrome” (thesis, University of Pittsburgh, Graduate School of Public Health, 2011).

[11] C. P. Torfs and R. E. Christianson , “Socioeconomic Effects on the Risk of Having a Recognized Pregnancy With Down syndrome,” Birth Defects Research Part A: Clinical and Molecular Teratology 67 (2003): 522–528, 10.1002/bdra.10071. 14565624

[12] L. R. Chapman , I. V. P. Ramnarine , D. Zemke , A. Majid , and S. M. Bell , “Gene Expression Studies in Down Syndrome: What Do They Tell Us About Disease Phenotypes?,” International Journal of Molecular Sciences 25 (2024): 2968, 10.3390/ijms25052968. 38474215 PMC10932069

[13] C. Do , Z. Xing , Y. E. Yu , and B. Tycko , “Trans‐Acting Epigenetic Effects of Chromosomal Aneuploidies: Lessons From Down syndrome and Mouse Models,” Epigenomics 9 (2017): 189–207, 10.2217/epi-2016-0138. 27911079 PMC5549717

[14] T. R. Oliver , E. Feingold , K. Yu , et al., “New Insights Into Human Nondisjunction of Chromosome 21 in Oocytes,” PLoS Genetics 4 (2008): e1000033, 10.1371/journal.pgen.1000033. 18369452 PMC2265487

[15] S. Ghosh , E. Feingold , and S. K. Dey , “Etiology of Down syndrome: Evidence for Consistent Association Among Altered Meiotic Recombination, Nondisjunction, and Maternal Age Across Populations,” American Journal of Medical Genetics. Part A 149 (2009): 1415–1420, 10.1002/ajmg.a.32932. PMC2732749 19533770

[16] T. R. Oliver , C. D. Middlebrooks , S. W. Tinker , et al., “An Examination of the Relationship Between Hotspots and Recombination Associated With Chromosome 21 Nondisjunction,” PLoS One 9 (2014): e99560, 10.1371/journal.pone.0099560. 24926858 PMC4057233

[17] M. A. Hultén , S. D. Patel , M. Tankimanova , et al., “On the Origin of Trisomy 21 Down syndrome,” Molecular Cytogenetics 1 (2008): 21, 10.1186/1755-8166-1-21. 18801168 PMC2564957

[18] P. Halder , U. Pal , A. Ganguly , et al., “Genetic Aetiology of Down syndrome Birth: Novel Variants of Maternal DNMT3B and RFC1 Genes Increase Risk of Meiosis II Nondisjunction in the Oocyte,” Molecular Genetics and Genomics 298 (2023): 293–313, 10.1007/s00438-022-01981-4. 36447056

[19] T. Renee Oliver , C. Middlebrooks , and A. Harden , “Variation in the Zinc Finger of PRDM9 Is Associated With the Absence of Recombination Along Nondisjoined Chromosomes 21 of Maternal Origin,” Journal of Down Syndrome & Chromosome Abnormalities 02 (2016): 02, 10.4172/2472-1115.1000115. PMC5502783 28702511

[20] U. Pal , P. Halder , A. Ray , et al., “The Etiology of Down syndrome: Maternal MCM9 Polymorphisms Increase Risk of Reduced Recombination and Nondisjunction of Chromosome 21 During Meiosis I Within Oocyte,” PLoS Genetics 17 (2021): e1009462, 10.1371/journal.pgen.1009462. 33750944 PMC8021012

[21] S. Ghosh , C. S. Hong , E. Feingold , et al., “Epidemiology of Down syndrome: New Insight Into the Multidimensional Interactions Among Genetic and Environmental Risk Factors in the Oocyte,” American Journal of Epidemiology 174 (2011): 1009–1016, 10.1093/aje/kwr240. 21957181

[22] E. G. Allen , S. B. Freeman , C. Druschel , et al., “Maternal Age and Risk for Trisomy 21 Assessed by the Origin of Chromosome Nondisjunction: A Report From the Atlanta and National Down syndrome Projects,” Human Genetics 125 (2009): 41–52, 10.1007/s00439-008-0603-8. 19050929 PMC2833410

[23] S. L. Sherman , S. B. Freeman , E. G. Allen , and N. E. Lamb , “Risk Factors for Nondisjunction of Trisomy 21,” Cytogenetic and Genome Research 111 (2005): 273–280, 10.1159/000086900. 16192705

[24] S. Tekin , A. Ocal , F. Y. Guleroglu , et al., “Oxidative Stress Biomarkers as Novel Screening Tools for Trisomy 21: a Case‐Control Study,” BMC Pregnancy and Childbirth 25 (2025): 578, 10.1186/s12884-025-07601-4. 40380151 PMC12084962

[25] T. Hassold , S. Sherman , and P. Hunt , “Counting Cross‐Overs: Characterizing Meiotic Recombination in Mammals,” Human Molecular Genetics 9 (2000): 2409–2419, 10.1093/hmg/9.16.2409. 11005796

[26] I. Scala , B. Granese , M. Sellitto , et al., “Analysis of Seven Maternal Polymorphisms of Genes Involved in Homocysteine/Folate Metabolism and Risk of Down Syndrome Offspring,” Genetics in Medicine 8 (2006): 409–416, 10.1097/01.gim.0000228206.21793.82. 16845273

[27] F. Ciccarone , E. Valentini , M. Malavolta , et al., “DNA Hydroxymethylation Levels Are Altered in Blood Cells From Down syndrome Persons Enrolled in the MARK‐AGE Project,” Journals of Gerontology: Series A 73 (2018): 737–744, 10.1093/gerona/glx198. PMC5946825 29069286

[28] M. Mendioroz , C. Do , X. Jiang , et al., “Trans Effects of Chromosome Aneuploidies on DNA Methylation Patterns in Human Down syndrome and Mouse Models,” Genome Biology 16 (2015): 263, 10.1186/s13059-015-0827-6. 26607552 PMC4659173

[29] L. Wan , Y. Li , Z. Zhang , Z. Sun , Y. He , and R. Li , “Methylenetetrahydrofolate Reductase and Psychiatric Diseases,” Translational Psychiatry 8 (2018): 242, 10.1038/s41398-018-0276-6. 30397195 PMC6218441

[30] M. Tsutsumi , R. Fujiwara , H. Nishizawa , et al., “Age‐Related Decrease of Meiotic Cohesins in Human Oocytes,” PLoS One 9 (2014): e96710, 10.1371/journal.pone.0096710. 24806359 PMC4013030

[31] P. A. Hunt , K. E. Koehler , M. Susiarjo , et al., “Bisphenol A Exposure Causes Meiotic Aneuploidy in the Female Mouse,” Current Biology 13 (2003): 546–553, 10.1016/S0960-9822(03)00189-1. 12676084

[32] A. Hale and G. L. Moldovan , “Novel Insights Into the Role of Bisphenol A (BPA) in Genomic Instability,” NAR Cancer 6 (2024): 6, 10.1093/narcan/zcae038. PMC11420844 39319028

[33] J. P. Corder , F. J. S. Al Ahbabi , H. S. Al Dhaheri , and F. Chedid , “Demographics and Co‐Occurring Conditions in a Clinic‐Based Cohort With Down syndrome in the United Arab Emirates,” American Journal of Medical Genetics. Part A 173 (2017): 2395–2407, 10.1002/ajmg.a.38338. 28686324

[34] A. Ray , T. R. Oliver , P. Halder , et al., “Risk of Down Syndrome Birth: Consanguineous Marriage Is Associated With Maternal Meiosis‐II Nondisjunction at Younger Age,” American Journal of Medical Genetics. Part A 176 (2018): 2342–2349, 10.1002/ajmg.a.40511. 30240118

[35] C. Keen , J. E. Hunter , E. G. Allen , C. Rocheleau , M. Waters , and S. L. Sherman , “The Association Between Maternal Occupation and Down Syndrome: A Report From the National Down Syndrome Project,” International Journal of Hygiene and Environmental Health 223 (2020): 207–213, 10.1016/j.ijheh.2019.09.001. 31519426

[36] S. L. Sherman , E. G. Allen , L. H. Bean , and S. B. Freeman , “Epidemiology of Down Syndrome,” Mental Retardation and Developmental Disabilities Research Reviews 13 (2007): 221–227, 10.1002/mrdd.20157. 17910090

[37] A. R. Savage , M. B. Petersen , D. Pettay , et al., “Elucidating the Mechanisms of Paternal Non‐Disjunction of Chromosome 21 in Humans,” Human Molecular Genetics 7, no. 8 (1998): 1221–1227, 10.1093/hmg/7.8.1221. 9668162

[38] F. Zhou , J. Ren , X. Lu , S. Ma , and C. Wu , “Gene‐Environment Interaction: A Variable Selection Perspective,” Epistasis: Methods and Protocols (2021): 191–223, 10.1007/978-1-0716-0947-7_13. 33733358

[39] S. Kumar , and K. Selvakumar , 2024, “Down Syndrome: Insights and Analysis,” Archives of Molecular Biology and Genetics 3 (2024): 24–27, 10.33696/genetics.3.017.

[40] A. F. Mentis , “Epigenomic Engineering for Down syndrome,” Neuroscience & Biobehavioral Reviews 71 (2016): 323–327, 10.1016/j.neubiorev.2016.09.012. 27646312

[41] M. Hetman and E. Barg , “Pediatric Population With Down syndrome: Obesity and the Risk of Cardiovascular Disease and Their Assessment Using Omics Techniques—Review,” Biomedicines 10 (2022): 3219, 10.3390/biomedicines10123219. 36551975 PMC9775395

[42] A. Asim , A. Kumar , S. Muthuswamy , S. Jain , and S. Agarwal , “Down Syndrome: An Insight of the Disease,” Journal of Biomedical Science 22 (2015): 41, 10.1186/s12929-015-0138-y. 26062604 PMC4464633

[43] P. Girchenko , J. Lahti , D. Czamara , et al., “Associations Between Maternal Risk Factors of Adverse Pregnancy and Birth Outcomes and the Offspring Epigenetic Clock of Gestational Age at Birth,” Clinical Epigenetics 9 (2017): 49, 10.1186/s13148-017-0349-z. 28503212 PMC5422977

[44] A. M. Moore , Z. Xu , R. T. Kolli , A. J. White , D. P. Sandler , and J. A. Taylor , “Persistent Epigenetic Changes in Adult Daughters of Older Mothers,” Epigenetics 14 (2019): 467–476, 10.1080/15592294.2019.1595299. 30879397 PMC6557560

[45] J. E. Hunter , E. G. Allen , M. Shin , et al., “The Association of Low Socioeconomic Status and the Risk of Having a Child With Down Syndrome: A Report From The National Down Syndrome Project,” Genetics in Medicine 15 (2013): 698–705, 10.1038/gim.2013.34. 23558253 PMC4122862

[46] G. Dominguez , Y. Wu , and J. Zhou , “Epigenetic Regulation and Neurodevelopmental Disorders: From MeCP2 to the TCF20/PHF14 Complex,” Genes 15 (2024): 1653, 10.3390/genes15121653. 39766920 PMC11728296

[47] A. M. Koul , F. Ahmad , A. Bhat , et al., “Unraveling Down Syndrome: From Genetic Anomaly to Artificial Intelligence‐Enhanced Diagnosis,” Biomedicines 11 (2023): 3284, 10.3390/biomedicines11123284. 38137507 PMC10741860

[48] A. Banik , D. Kandilya , S. Ramya , W. Stünkel , Y. Chong , and S. Dheen , “Maternal Factors That Induce Epigenetic Changes Contribute to Neurological Disorders in Offspring,” Genes 8 (2017): 150, 10.3390/genes8060150. 28538662 PMC5485514

[49] P. Halder , U. Pal , A. Ganguly , et al., “Understanding Etiology of Chromosome 21 Nondisjunction From Gene × Environment Models,” Scientific Reports 11 (2021): 22390, 10.1038/s41598-021-01672-x. 34789805 PMC8599692

[50] J. Aprigio , C. M. L. de Castro , M. A. C. Lima , M. G. Ribeiro , I. M. Orioli , and M. R. Amorim , “Mothers of Children With Down syndrome: A Clinical and Epidemiological Study,” Journal of Community Genetics 14 (2023): 189–195, 10.1007/s12687-022-00627-7. 36562914 PMC10104982

[51] N. Gensous , C. Franceschi , S. Salvioli , P. Garagnani , and M. G. Bacalini , “Down Syndrome, Ageing and Epigenetics,” vol. 91, (Springer, 2019), 161–193, 10.1007/978-981-13-3681-2_7. 30888653

[52] L. Udry‐Jørgensen , J. Darwiche , M. Germond , D. Wunder , and Y. Vial , “Anxiety, Depression, and Attachment Before and After the First‐Trimester Screening for Down syndrome: Comparing Couples Who Undergo Art With Those Who Conceive Spontaneously,” Prenatal Diagnostics 35 (2015): 1287–1293, 10.1002/pd.4688. 26348779

[53] A. Reece and G. Hulse , “Socioeconomic, Ethnocultural, Substance‐ and Cannabinoid‐Related Epidemiology of Down Syndrome USA 1986–2016: Combined Geotemporospatial and Causal Inference Investigation,” International Journal of Environmental Research and Public Health 19 (2022): 13340, 10.3390/ijerph192013340. 36293924 PMC9602855

[54] S. Ghosh , P. Ghosh , and S. K. Dey , “Altered Incidence of Meiotic Errors and Down syndrome Birth Under Extreme Low Socioeconomic Exposure in the Sundarban Area of India,” Journal of Community Genetics 5 (2014): 119–124, 10.1007/s12687-013-0159-8. 23857082 PMC3955464

[55] S. E. Antonarakis , B. G. Skotko , M. S. Rafii , et al., “Down Syndrome,” Nature Reviews Disease Primers 6 (2020): 9, 10.1038/s41572-019-0143-7. PMC8428796 32029743

[56] D. Mazurek , J. Wyka , D. Mazurek , and J. Wyka , “Down Syndrome‐‐Genetic and Nutritional Aspects of Accompanying Disorders,” Roczniki Państwowego Zakładu Higieny 66, no. 3 (2015): 189–194. 26400113

[57] S. Horvath , P. Garagnani , M. G. Bacalini , et al., “Accelerated Epigenetic Aging in Down Syndrome,” Aging cell 14 (2015): 491–495, 10.1111/acel.12325. 25678027 PMC4406678

